# Implementation of guidelines on Family Involvement for persons with Psychotic disorders: a pragmatic cluster randomized trial. Effect on relatives’ outcomes and family interventions received

**DOI:** 10.3389/fpsyt.2024.1381007

**Published:** 2024-05-24

**Authors:** Irene Norheim, Reidar Pedersen, Maria Lie Selle, Jan Ivar Røssberg, Lars Hestmark, Kristin Sverdvik Heiervang, Torleif Ruud, Vilde Maria Åsholt, Kristiane Myckland Hansson, Paul Møller, Roar Fosse, Maria Romøren

**Affiliations:** ^1^ Department of Mental Health Research and Development, Division of Mental Health and Addiction, Vestre Viken Hospital Trust, Drammen, Norway; ^2^ Centre for Medical Ethics, Institute of Health and Society, University of Oslo, Oslo, Norway; ^3^ Health Services Research Unit, Akershus University Hospital, Lørenskog, Norway; ^4^ Section for Treatment Research, Division of Mental Health and Addiction, Oslo University Hospital, Oslo, Norway; ^5^ Institute of Clinical Medicine, Faculty of Medicine, University of Oslo, Oslo, Norway; ^6^ Division of Mental Health Services, Akershus University Hospital, Lørenskog, Norway

**Keywords:** schizophrenia, severe mental illness, caregivers, family intervention, family psychoeducation, evidence based practices, mental health services

## Abstract

**Background:**

Family interventions (FI) are recommended as part of the treatment for psychotic disorders, but the implementation in mental health services is generally poor. Recently, The Implementation of guidelines on Family Involvement for persons with Psychotic disorders (IFIP) trial, demonstrated significant improvements in implementation outcomes at cluster-level. This sub-study aims to examine the effectiveness of the IFIP intervention on relatives’ outcomes and received FI.

**Methods:**

A cluster randomized controlled trial, was conducted in 15 Norwegian Community Mental Health Center (CMHC) units that were randomized to either the IFIP intervention, including implementation interventions and clinical interventions, or treatment as usual (TAU). The clinical interventions consisted of FI: basic family involvement and support (BFIS) to all patients and family psychoeducation (FPE) to as many as possible. Patients with psychotic disorders and their closest relative were invited to fill in questionnaires at inclusion and 6 months and 12 months follow-up. Received FI was reported by both relatives and clinicians. The relatives’ primary outcome was satisfaction with health service support, measured by the Carer well-being and support questionnaire part B (CWS-B). The relatives’ secondary outcomes were caregiver experiences, expressed emotions and quality of life. Patients’ outcomes will be reported elsewhere.

**Results:**

In total 231 patient/relative pairs from the CMHC units were included (135 intervention; 96 control).The relatives in the intervention arm received an increased level of BFIS (p=.007) and FPE (p < 0.05) compared to the relatives in the control arm, including involvement in crisis planning. The primary outcome for relatives’ satisfaction with health service support, showed a non-significant improvement (Cohen’s d = 0.22, p = 0.08). Relatives experienced a significant reduced level of patient dependency (Cohen’s d = -0.23, p = 0.03).

**Conclusion:**

The increased support from clinicians throughout FI reduced the relatives’ perceived level of patient dependency, and may have relieved the experience of responsibility and caregiver burden. The COVID-19 pandemic and the complex and pioneering study design have weakened the effectiveness of the IFIP intervention, underscoring possible potentials for further improvement in relatives’ outcomes.

**Clinical Trial Registration:**

ClinicalTrials.gov, identifier NCT03869177.

## 1 Introduction

Psychotic disorders often lead to long-lasting functional impairment, affecting the life and well-being of both patients (1) and relatives (2, 3). Evidence based treatment include psychosocial and pharmacological interventions (4, 5). One of the most efficacious and best researched psychosocial treatments is structured family interventions (FI), such as family psychoeducation (FPE) (6). FI can decrease relapses, reduce hospital admissions, improve medication adherence, and improve general social functioning and levels of expressed emotion within the family (6–12). Studies of FI also indicate a reduction in global disease burden, negative caregiving experiences, and perceived burden among relatives (13–17).

Relatives may provide unique and long-lasting social support, while being a rehabilitation resource for patients with psychotic disorders. At the same time, the nature of psychosis constitutes a great burden for relatives and may lead to depression (18), stress and burnout for the next-of-kin (19). Family involvement may therefore be a crucial part of treatment, aiming to improve the conditions for both patients’ and relatives’ psychosocial health and well-being. FI is consistently recommended in treatment guidelines worldwide at all stages of psychotic disorders (20–24) but is too often not provided in routine clinical practice (25–27). It is also well known that even basic family involvement, to meet the relative’s needs for information and guidance (28) is both of varying quality and inconsistently provided in adult mental health services and rarely reaches more than 20 – 30% of the patients (27, 29–33). Various factors at different levels can explain why attempts to implement family interventions in routine care have failed. Many are related to multiple implementation barriers to family involvement in particular, others are more general barriers to translating evidence into everyday clinical practice. As a result, the family often play an active role in caring for the patient, to ensure information and to be involved in care and treatment, and some are also plainly rejected by the services (34–36).

Norwegian national guidelines recommend FPE as a first-line treatment for people with psychotic disorders (24), and basic family involvement practices in the health services in general (37). Based on the evidence of poor implementation and the lack of knowledge about family involvement in naturalistic clinical settings (38), we carried out a large-scale non-blind pragmatic cluster randomized controlled trial (c-RCT), *The Implementation of guidelines on Family Involvement for persons with Psychotic disorders (IFIP) trial* (39) in Community Mental Health Centers (CMHC). The purpose of the IFIP trial was to improve implementation of the national guidelines for the benefit of patients and their relatives. The study combined implementation outcomes at the level of CMHC units and effectiveness outcomes for the included patients and their relatives. The IFIP trial’s implementation outcome study showed that it was possible to significantly improve both the quality and quantity of basic family involvement and FPE in ordinary services on a large scale (40).

To our knowledge, the IFIP trial is the first randomized trial investigating the effectiveness of a large-scale FI implementation support program in ordinary mental health services for patients with psychotic disorders. This kind of multi-level, large scale naturalistic studies have been recommended for years (41), but are still quite rarely performed. Such studies are likely to be of particular importance in areas with large evidence-to-practice gaps, as is definitely the case when it comes to FI in the treatment of psychotic disorders. We have identified only one previous cluster randomized implementation study that also reported patients’ and relatives’ outcomes, in which FI was evaluated together with several other types of treatment interventions and only for first-episode psychosis (42, 43). The study showed enhanced clinical status and social functioning in the patients, along with reduced burden and improved emotional distress, as well as increased satisfaction with the services for the relatives.

The present effectiveness study examines differences in outcomes between the included relatives in the intervention and control arm by answering the following research questions: 1. What was the type and amount of FI received among the relatives in the intervention arm compared to those in the control arm? 2. Did relatives in the intervention arm experience an improvement in satisfaction with health service support, caregiver experiences, expressed emotions and quality of life compared to those in the control arm? Patient outcomes will be reported elsewhere.

## 2 Methods

This article conforms to the “Consolidated Standards of Reporting Trials (CONSORT) statement 2010: extension to cluster randomized trials” (44) (**Supplementary File 1**). The study protocol has been published elsewhere (39), but the study design and interventions are briefly described below.

### 2.1 Trial design, participants and procedures

The IFIP trial included 15 psychiatric outpatient clinics from 12 CMHCs in five Health Trusts in South-Eastern Norway, which together covered a catchment area of 1.3 million inhabitants, including both urban and rural populations (27). Each of the 15 CMHC units represented one cluster, except two units that were merged, resulting in 14 clusters (45). A detailed account of the 15 participating CMHC units (27) and their randomization into 14 clusters have been published (40). The units of analysis in the current study were relatives.

The participating CMHC units were asked to include patients with a psychotic disorder together with their closest relative in pairs during the recruitment period from March 2019 to September 2020. Patients were invited to participate in the study, and those who agreed to participate provided written informed consent to participate and for the clinician to contact a close relative for participation. Subsequently, relatives were contacted and invited to participate, and consenting relatives provided written informed consent. At the study outset, each CMHC unit had between 28 and 217 patients with psychotic disorders, with 1392 patients in total. Patients and relatives were recruited in pairs to explore and compare independent and dependent variables from both respondents. Patient results and dyadic results will be published elsewhere.

The inclusion criteria for patients were: having an established or tentative psychotic disorder (F20–29 in ICD-10) certain enough to begin treatment; being 18 years or older. The diagnosis was made by local clinicians. The exclusion criteria for patients were: being a forensic client; not being competent to consent to participation; not having any relatives; having previously completed more than five sessions of FPE in single-family groups or more than ten sessions of FPE in multi-family groups or a similarly structured FI. Inclusion criteria for relatives were: being a relative of a patient with a diagnosis as described above; being 18 years or older.

The respondents were recruited independent of the decision to offer FI or other treatments. This means that those recruited did not have to undergo any specific treatment, intervention, or support during the follow-up period, in either the intervention or control arm. Similarly, there was no obligation for patients and relatives who received FI, to participate in the study. This separation of research from treatment had an ethical purpose of avoiding favoritism towards participants with better treatment. Additionally, there was an academic rationale: to examine the effectiveness of improved practices of family involvement in clinical units on a broader group of patients and relatives, not just those who received a specific intervention (39).

The intervention period started in February 2019 with a half-day “kick-off” about the significance of family involvement in all the intervention arm units, and subsequently a one week FPE course was arranged. During the intervention period the control arm units provided treatment as usual (TAU). Research activities for the IFIP trial are seen in **Supplementary File 2**.

### 2.2 The IFIP intervention

Preliminary mapping in the participating CMHC units showed that the proportion of patients who received basic family involvement practices was 20–40%, while for FPE it was 4% (27). The purpose of the IFIP intervention was to establish, improve and maintain basic family involvement practices and FPE as routine treatment, through a set of implementation interventions, and thereby improve outcomes for the patients and their relatives. The IFIP intervention was based on two levels of family involvement applicable for patients with psychotic disorders and their relatives in Norway: Basic family involvement practices for the health- and care services in general based on legal regulations, research evidence, ethical considerations, and discussions between key stakeholders and experts (37), FPE as first-line treatment in the national guidelines for persons with psychotic disorders. The latter is based on a systematic review and quality assessment of relevant literature, and professional, experience-based, and contextual considerations made by the parties involved in the work (24). The approach for the clinical interventions was that clinicians should provide FI consisting of basic family involvement practices to all patients and their relatives, and FPE to as many as possible. The background and development of the IFIP intervention and the chosen implementation strategies, are described and discussed in detail elsewhere (39, 40).

#### 2.2.1 Clinical interventions

The recommendations for basic family involvement practices were operationalized and co-adapted in collaboration with panel groups of patients, relatives and clinicians and developed into The Basic Family Involvement and Support (BFIS) intervention (40) consisted of: 1) at least three conversations about family involvement (one with the patient, one with the relative(s), and one with patient and relative(s) together), 2) written information about the unit’s family involvement, relevant web resources and municipal- and peer support services, and 3) psychoeducative seminars for relatives on relevant topics. The purpose of the BFIS was to ensure conversations about the importance of involving the family in the treatment, offering FPE, providing information about psychotic disorders and preparing a crisis plan. The FPE consisted of single-family groups with separate alliance sessions, followed by joint sessions with the patient and the relative(s) together for 12 months, or adapted to the family’s needs, based on McFarlane’s family work (46). FPE comprises of sharing knowledge about psychosis and its treatment, while improving the family’s coping strategies through crisis management, as well as communication and problem-solving skills. Preparing a crisis plan was recommended in both the clinical interventions and is a type of advance statement that describes how to recognize early signs of mental crisis and how these can be managed (47). The purpose of providing BFIS in addition to FPE was to ensure that all relatives received a minimum level of family involvement. BFIS required less time and had a lower threshold for clinicians to initiate than FPE and could therefore serve as a first step in family involvement through FPE.

#### 2.2.2 Implementation interventions

The IFIP intervention included selected implementation interventions to strengthen and assist the CMHC units’ implementation of the clinical interventions: 1) a family coordinator within each unit to coordinate and sustain family involvement and support, 2) an implementation team within each unit to supervise the implementation process with assistance from the project, 3) training and supervision of all clinicians in important aspects of family involvement, including kick-off, FPE course and network conferences, 4) shared resources like lectures, information leaflets, and procedures, and 5) fidelity measurements of adherence to the guidelines, with tailored on-site feedback and supervision.

#### 2.2.3 Implementation strategies

The chosen implementation strategies consisted of 1) leadership commitment, 2) stakeholder engagement inspired by a responsive evaluation approach (48), and 3) a “whole-ward approach” intended to change the culture and clinical way of working (49).

Due to the COVID-19 pandemic, people with psychosis and their relatives experienced reduced access to health and care services in Norway (50, 51). There was a problem with providing the clinical interventions after the lock-down in Norway from 12^th^ of March 2020 (**Supplementary File 2**). To uphold the health care services, the Norwegian Government urged the services to make use of digital services including video consultations. Some of the participating units adapted to these changes, while the majority struggled due to the sum of restrictions, lack of staff and problems with the digital adjustments. Depending on the different phases of the pandemic, clinicians had to adjust and change the way they provided BFIS and FPE. As a consequence, the clinical interventions were interrupted, put on hold, or postponed.

### 2.3 Outcome measures

As background variables we collected a range of sociodemographic data from the patients and the relatives (e.g. age, sex, civil status) and the patients’ level of functioning measured with the Global Assessment of Functioning (GAF) scale (52). Relatives’ outcomes were assessed through self-reported questionnaires. Outcome of interest was defined as difference from baseline to 6 and 12 months follow-up. The rationale behind the choice of outcomes and full details of all measures are described in the IFIP study protocol (39).

#### 2.3.1 Primary outcome

The primary outcome for the relatives was satisfaction with health service support. This was assessed using the Carer well-being and support questionnaire version 2, part B - support scale (CWS-B) (53).

#### 2.3.2 Secondary outcomes

The secondary outcomes for the relatives were experience of caregiving, expressed emotions, quality of life, and family involvement and support services received. Perceived experience of caregiving was assessed using The Experience of Care-giving inventory questionnaire (ECI) (54). Expressed emotion was assessed using The Family questionnaire (FQ) (55). Quality of life was assessed using The Care Related Quality of Life questionnaire (CarerQoL) (56). The CarerQol instrument combines a description of the caregiving situation (CarerQol-7D) with a valuation component in terms of general well-being (CarerQol-VAS, Visual Analogue Scale).

Service level outcomes were measured by clinicians at baseline and 12 months follow-up in terms of number of medical record-reported FPE sessions. In addition, relatives reported their participation in any family involvement and support services, in or outside the CMHC units. At baseline, the relatives reported such participation “the last 12 months” and also participation before that, whereas at 6 and 12 months follow-up, only “participation the last 6 months” was reported.

### 2.4 Sample size

Power analyses were performed for the satisfaction with health service support with CWS-B (53). Seven clusters in each arm and a sample size of 112 relatives per arm was needed to detect an increase of 0.5 SD from baseline to 12 months, with 80% power and 5% two-tailed significance level in a cluster-randomized trial with intra-cluster correlation coefficient (ICC) = 0.05 (45, 57).

### 2.5 Statistical analyses

For the first research question, the number and percentages of each type of family involvement and support services for relatives were calculated and compared between the intervention and the control arm using the chi-squared test. Some relatives had already received a certain level of services at baseline (in accordance with the inclusion criteria). This occurred because the recruitment of participants took place concurrently with the implementation of the clinical interventions (**Supplementary File 2**). Additionally, various types of family involvement and support occurred independently of the IFIP trial (before, during, and after) in both arms due to the naturalistic study design. Because of this, the category “previous participation” was included in the analysis where this had been collected.

For the second research question, means, standard deviations, and number of observations for all scores were described. For the primary outcomes of interest, CWS-B for relatives, we estimated linear mixed effects models estimating the mean outcome in each treatment arm at 0, 6 and 12 months. The fixed part of the model consisted of intercept, treatment arm, dummy variables for time at 6 and 12 months, and interaction terms between treatment arm and dummy variables for time. The random part of the model consisted of random intercept for relative identification nested within center. If the ICC for center was negligible, the random part of the model was reduced to random intercept for relative identification. We included only relatives with at least one follow-up measurement (6 months, 12 months, or both). Missing data were handled by multiple imputation using predictive mean matching with 30 imputed data sets (58). The imputation model included age and sex in addition to the treatment arm, center, and any previous scores. For selected secondary outcomes, we applied the same model as for the primary outcome.

The results were reported as regression coefficients, standard errors, 95% confidence intervals (CI) and p-values. We further reported the estimated differences in change with time between the two arms together with 95% CI, effect sizes (Cohen’s d) and p-values. For the primary outcome we illustrated graphically the estimated outcomes at each time point with 95% CI. All tests were two-sided with a significance level of 0.05, and residual diagnostics were conducted by assessing the residuals graphically. No adjustment for multiple testing was implemented, and we present the crude p-values for the reader to interpret. In all the mixed effects model analyses, we used R version 3.4.4 (lmer-functions).

### 2.6 Sensitivity analyses

The models were rerun on a subset of the data with complete outcomes at all three time points (complete case analysis). The regression coefficients from the complete case analysis (data not shown) were in similar magnitude and the same direction as the results from the main analysis with relatives with at least one follow-up presented above.

Baseline characteristics of the participants who were lost to follow-up, and therefore excluded from the analysis, were compared to the included relatives (with at least one follow-up) with respect to age and sex, ECI positive and negative subscale and CarerQoL-VAS. For relatives in the control arm, there were more males in the subgroup with no follow-ups (N = 10, prop males 60%), compared to those with at least one follow-up (N = 86, prop males 24%), (Fisher’s exact,p = 0.03). Relatives in the intervention arm with no follow-up were more happy (N=10, CarerQol-VAS 7.80 (SD = 1.90)) than those with at least one follow-up (N = 124, CarerQoL-VAS 6.48 (SD = 1.75)), (Wilcox rank sum, W = 362, p = 0.03).

### 2.7 Subgroup analyses

In order to measure the effectiveness of the clinical interventions directly we compared the following subgroups: 1. Relatives in the intervention arm receiving FPE the last 12 months after inclusion (N=29), 2. Relatives in the control arm not receiving FPE the last 12 months before inclusion (N=94). The linear mixed effects models for the subgroup analysis were adjusted for the patient’s duration of psychotic disorder and GAF-F and for the relative’s civil status, if they had higher education, and if they were living with the patient.

## 3 Results

### 3.1 Participants

**Figure 1:**
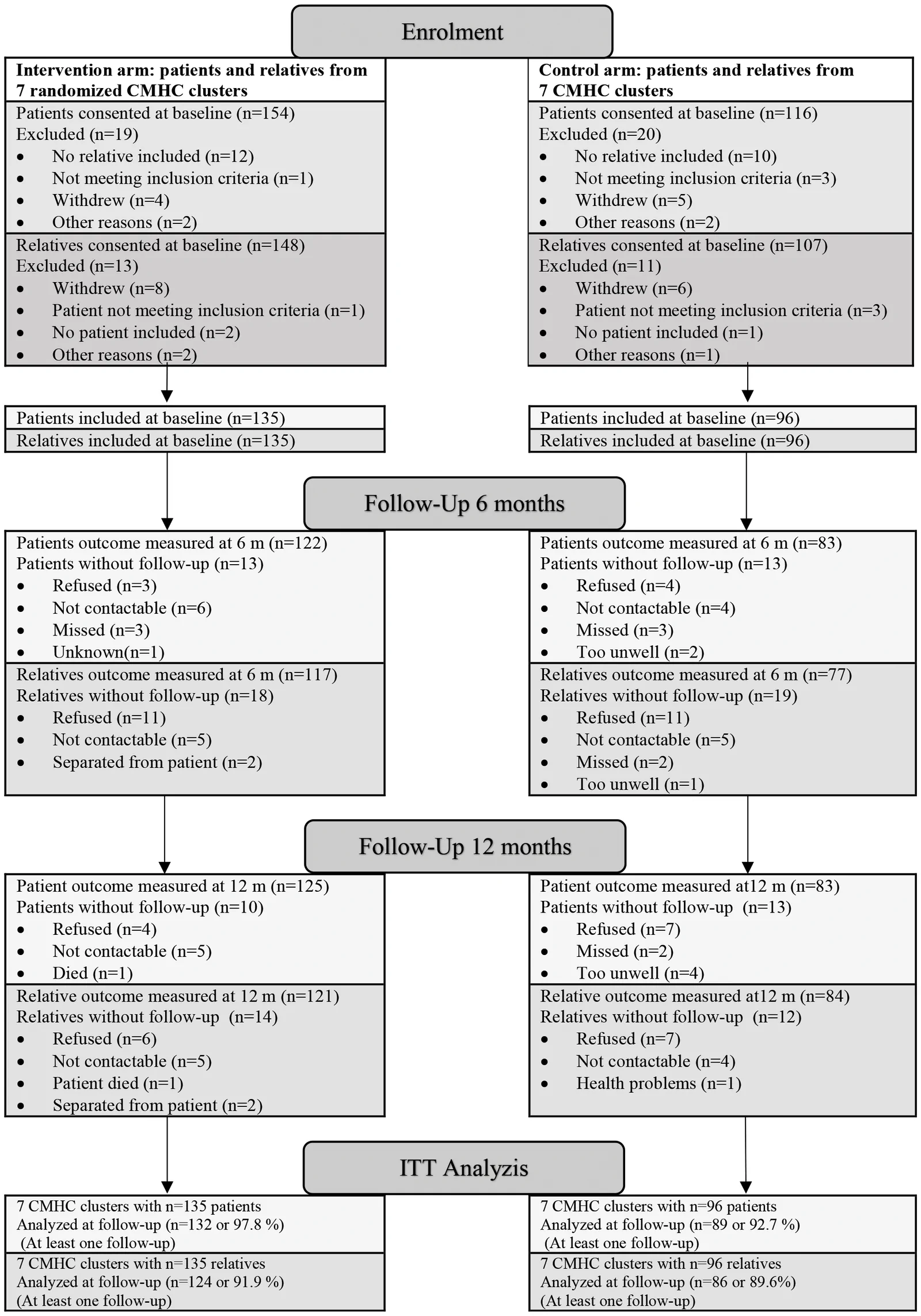
Consort diagram.

In total 261 patients (150 intervention; 111 control) and 239 relatives (139 intervention; 100 control) from the 14 randomized clusters consented for the trial. This paper presents the analysis from the relatives in 231 patient/relative pairs (135 intervention; 96 control) (**Figure 1**. Consort diagram).

Sociodemographic characteristics for patients and relatives, and the patients’ level of functioning and symptoms measured with GAF are presented in **Table 1**.

**Table 1:** Sociodemographic characteristics for relatives and patients at baseline.

Characteristics	Intervention (n = 135) f (%)	Control (n = 96) f (%)	Chi-square (χ^2^), df	P
Relatives				
Age	(1 missing)		-1.36,228	0.17
	M =53.5, SD=12.4	M =55.6, SD=14.3		
Gender	(1 missing)	(1 missing)	1.32,1	0.25
Male	49 (36.6)	27 (28.4)		
Female	85 (63.4)	68 (71.6)		
Civil status	(2 missing)	(1 missing)	5.84, 2	0.053
Single	13 (9.8)	11 (11.6)		
Married/co-habiting	101 (75.9)	59 (62.1)		
Divorced/separated/widowed	19 (14.3)	25 (26.3)		
Educational level	(2 missing)	(1 missing)	<0.01,1	0.964
Secondary school or less	59 (44.4)	41 (41.7)		
Higher education	74 (55.7)	54 (56.8)		
Working status	(2 missing)	(2 missing)	0.41,1	0.52
Unemployed or retired	47 (35.3)	38 (40.4)		
Employed	86 (64.7)	56 (59.5)		
Relation to the patient	(1 missing)	(1 missing)	Fisher exact	0.09
Parent	85 (63.4)	66 (69.5)		
Child	5 (3.7)	10 (10.5)		
Sibling	17 (12.7)	8 (8.4)		
Married/co-habiting	24 (17.9)	9 (9.5)		
Other	3 (2.2)	2 (2.1)		
Living with the patient	(1 missing)	(1 missing)	1.45,1	0.23
Yes	48 (35.8)	26 (27.4)		
Patients				
Age	M =35.1, SD=11.4	M=36.8, SD=13.7	T = -1.03, 229	0.30
Gender			0.10, 1	0.75
Male	80 (59.3)	54 (56.3)		
Female	55 (40.7)	42 (43.8)		
Civil status			2.58, 2	0.28
Single	95 (70.4)	74 (77.1)		
Married/co-habiting	31 (23.0)	14 (14.6)		
Divorced, separated, widowed	9 (6.7)	8 (7.1)		
Living arrangement	(1 missing)	(1 missing)	<0.01, 1	0.96
Alone	75 (56.0)	52 (54.7)		
With someone else	59 (44.0)	43 (45.3)		
Caring for children	(1 missing)		0.04, 1	0.83
Yes	12 (9.0)	7 (7.3)		
Main activity the last 6 month			11.49,2	0.003
Unemployed	75 (55.6)	48 (50)		
Employed	19 (14.1)	3 (3.1)		
Other	41 (30.4)	45 (46.9)		
Diagnosis			3.01, 2	0.22
Core schizophrenia (F20,21,22,25)	109 (80.7)	83 (86.5)		
Acute and transient psychosis (F22)	10 (7.4)	8 (8.3)		
Other psychosis (F28, 29)	16 (11.9)	5 (5.2)		
Age when diagnosed	M=26.6, SD=7.4	M=27.5, SD=10.2	T = -0.77, 229	0.44
GAF functioning	(9 missing)	(9 missing)	T = 0.05, 211	0.96
GAF-F	M=51.54, SD=13.04	M=51.45, SD=12.06		
GAF symptoms	(8 missing)	(9 missing)	T = 0.22, 212	0.82
GAF-S	M=52.57, SD=13.21	M=52.18, SD =10.65		

Before the COVID-19 pandemic lockdown, 26% of the relatives had answered the 6-months follow-up questionnaire. The remaining data for the 6- and 12-months follow-up were collected during the pandemic.

### 3.2 Effectiveness of the IFIP intervention on received family interventions

The proportion of patient/relative pairs in the intervention arm who had received FPE from the CMHC units at baseline was 17.2% (23/134), compared with 3% (3/96) in the control arm, and 37.9% (50/132) and 7% (6/86) respectively at 12 months follow-up. This increase in the intervention arm was significantly higher than the increase in the control arm, (p < 0.05). After the pandemic outbreak, only six new FPE groups in the intervention arm and two in the control arm were started.

**Table 2** shows the proportion of relatives who had received various types of family involvement and support services. The proportion of relatives who received at least one CMHC consultation “without the patient” had a larger increase from “previous” at baseline to the last 24 months at 12 months follow up in the intervention arm (from 27.6% to 64.2%) compared to the control arm (from 28.1% to 45.7%), (p < 0.05). At 24 months follow-up, the proportion of CMHC consultations “without the patient” and consultations “with patient” both were significantly higher in the intervention arm (64.2% and 72%) than in the control arm (45.7% and 53%), (p-values < 0.05).

**Table 2:** Descriptive analyzes of various type of family involvement and support services received by the relatives.

N relatives intervention (I) =135 N relatives control (C) = 96		Previous				24 months				Change previous vs 24 m	Interv. vs control	p-val Z-test
		n	N	%	p-val Chi-sq	n	N	%	p-val Chi-sq	Percen-tage points	Percen-tage points	
CMCH consultations												
Supportive/next of kin consultation without the patient	I	37	134	27,6		77	120	64,2		36,6		
	C	27	96	28,1	1	37	81	45,7	0,014	17,6	19,0	0,04
Supportive/next of kin consultation with the patient	I	38	134	28,4		87	121	72,0		43,5		
	C	21	96	21,9	0,338	44	83	53,0	0,009	31,1	12,4	0,165
Consultation with the patient’s clinician	I	32	134	23,9		99	128	77,3		53,5		
	C	23	96	24,0	1	56	85	65,9	0,092	41,9	11,5	0,176
Other support services												
Family therapy outside the CMHC unit	I	7	134	5,2		3	114	2,6		-2,6		
	C	8	96	8,3	0,42	1	75	1,3	1	-7,0	4,4	-
Services for relatives from the municipality	I	17	134	12,7		23	116	19,8		7,1		
	C	15	96	15,6	0,659	15	78	19,2	1	3,6	3,5	0,635
Peer support group for relatives	I	22	134	16,4		10	115	8,7		-7,7		
	C	18	96	18,8	0,777	8	76	10,5	0,864	-8,2	0,5	0,941
Seminars about mental disorders/being a relative	I	27	134	20,1		31	118	26,3		6,1		
	C	27	96	28,1	0,211	14	75	18,7	0,297	-9,5	15,6	0,062
Therapy due to the burden of being a relative	I	21	134	15,7		15	114	13,2		-2,5		
	C	19	96	19,8	0,524	17	78	21,8	0,168	2,0	-4,5	0,554
At least one of the services	I	75	134	56,0		118	129	91,5		35,5		
	C	58	96	60,4	0,591	74	88	84,1	0,146	23,7	11,8	0,142

**Figure 2:**
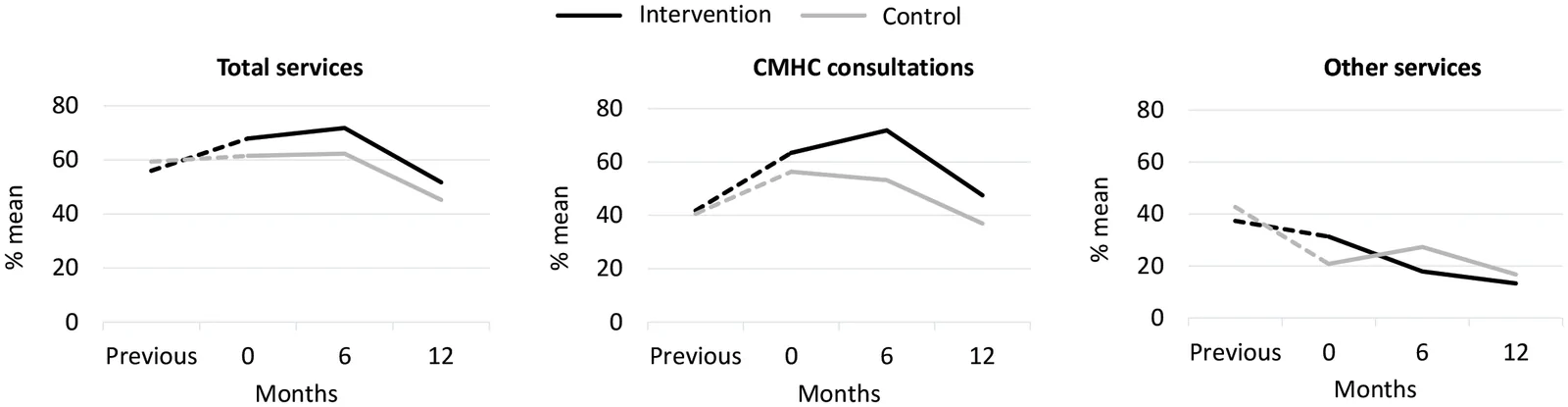
Proportion of relatives who received at least one consultation with Community mental health service (CMHC) or other services (e.g. municipalities, peer support organizations).

**Figure 2** shows that the proportion of relatives who had participated in at least one CMHC consultation in the intervention arm was highest at 6 months, before it was reduced at 12 months. The level of “other services” decreased in the intervention arm and increased in the control arm from baseline to 6 months.

In the intervention arm 79.8% (95/119) of the relatives had participated in a CMHC consultation during the one-year follow up, compared to 62.0% (49/79) in the control arm, (p = .007). The proportion of relatives who had participated in at least one CMHC consultation, at all time points (baseline, 6 and 12 months) did not differ significantly between the intervention arm (28.7%, 35/122) and the control arm (25.6%, 22/86).

Information and/or involvement of relatives in the patients’ crisis plan increased from 38.8% (52/134) at baseline to 55.3% (73/132) at 12 month in the intervention arm, and from 37.5% (36/96) to 38.8% (33/85) in the control arm.

### 3.3 Effectiveness of the IFIP intervention on relatives’ outcomes

#### 3.3.1 Primary outcome: satisfaction with health service support

**Table 3** describes the outcome scales, and presents the estimated difference in outcome change between the two arms from baseline to the follow-up time points. The primary outcome, relatives’ satisfaction with information, involvement and support from staff in their role as informal carers (sum-score CWS-B), had a higher increase in the intervention arm compared to the control arm at 12 months. This was however, a non-significant result (Cohen’s d = 0.22, p = 0.08).

**Table 3:** Estimated difference in outcome change between arms from baseline to follow-up, 95% confidence intervals, corresponding effect size (Cohen’s d) and p value.

				6 MONTHS FOLLOW-UP					12 MONTHS FOLLOW-UP				
Primary outcome	Item	L^1^	Range^2^	Exp-con	95% CI		Cohen’s d	P	Exp-con	95% CI		Cohen’s d	P
CWS Support sum	17	L-4	0–51^↑^	-0,26	-3,42	2,9	-0,02	0,87	2,79	-0,38	5,96	0,22	0,08
CWS Information and advice for carers	8	L-4	0–24^↑^	0,05	-1,53	1,63	0,01	0,95	1,09	-0,46	2,64	0,18	0,17
CWS Involvement in treatment and care planning	2	L-4	0–6^↑^	0,02	-0,32	0,35	0,01	0,92	0,29	-0,04	0,62	0,15	0,08
CWS Support from medical and/or care staff	7	L-4	0–21^↑^	-0,53	-2,1	1,05	-0,09	0,51	1,29	-0,32	2,91	0,23	0,12
Secondary outcomes	Item	L	Range	Exp-con	95% CI		Cohen’s d	P	Exp-con	95% CI		Cohen’s d	P
CWS additional question: Satisfaction^3^	1	L-4	0–3^↑^	-0,05	-0,35	0,25	-0,05	0,74	0,19	-0,11	0,5	0,19	0,21
ECI Negative scale (1.2.3.5.6.7.9.10)	52	L-5	0–208^↓^	-2,96	-9,03	3,11	-0,09	0,34	-3,26	-9,18	2,66	-0,09	0,28
ECI Positive scale (4.8)	14	L-5	0–56^↑^	-1	-2,74	0,75	-0,12	0,26	-0,65	-2,34	1,05	-0,08	0,45
1) ECI Difficult behaviors	8	L-5	0–32^↓^	-0,33	-1,71	1,04	-0,05	0,63	-0,31	-1,68	1,06	-0,05	0,65
2) ECI Negative symptoms	6	L-5	0–24^↓^	-0,74	-1,98	0,5	-0,13	0,24	-0,86	-2,05	0,33	-0,15	0,16
3) ECI Stigma	5	L-5	0–20^↓^	0,03	-0,8	0,85	0,01	0,95	0,05	-0,76	0,86	0,01	0,9
4) ECI Problems with services	8	L-5	0–32^↓^	0,05	-1,41	1,51	0,01	0,95	-0,58	-1,99	0,84	-0,09	0,42
5) ECI Effects on family	7	L-5	0–28^↓^	0,35	-0,73	1,43	0,06	0,52	0,25	-0,8	1,31	0,04	0,64
6) ECI Need to backup	6	L-5	0–24^↓^	-0,99	-2	0,01	-0,19	0,05	-0,44	-1,41	0,53	-0,09	0,37
7) ECI Dependency	5	L-5	0–20^↓^	-0,81	-1,67	0,05	-0,19	0,06	-0,96	-1,81	-0,11	-0,23	**0,03**
8) ECI Loss	7	L-5	0–28^↓^	-0,38	-1,37	0,61	-0,07	0,45	-0,48	-1,44	0,48	-0,09	0,32
9) ECI Positive personal experiences	8	L-5	0–32^↑^	-0,98	-2,15	0,19	-0,18	0,1	-0,52	-1,67	0,63	-0,09	0,37
10) ECI Good aspects of relationship	6	L-5	0–24^↑^	-0,11	-1,02	0,79	-0,03	0,81	-0,11	-0,99	0,78	-0,03	0,81
FQ Emotional overinvolvement	10	L-4	10–40^↓^	0,81	-0,26	1,89	0,15	0,14	0,32	-0,71	1,35	0,06	0,55
FQ Criticism	10	L-4	10–40^↓^	0,19	-0,99	1,37	0,03	0,75	0,27	-0,88	1,43	0,05	0,64
CarerQoL-VAS	1	L-10	0–10^↑^	0,19	-0,22	0,6	0,11	0,36	0,2	-0,2	0,61	0,11	0,33

^1^L=Likert scale: Numbers are number of points in the scale. ^2^Range: ^↓^= Higher value, negative outcome, ^↑^= Higher value, positive outcome. ^3^A single question about satisfaction with health services from an earlier version of CWS-B: Overall, how satisfied are you with the support you receive to help you in your role as a relative?

**Figure 3:**
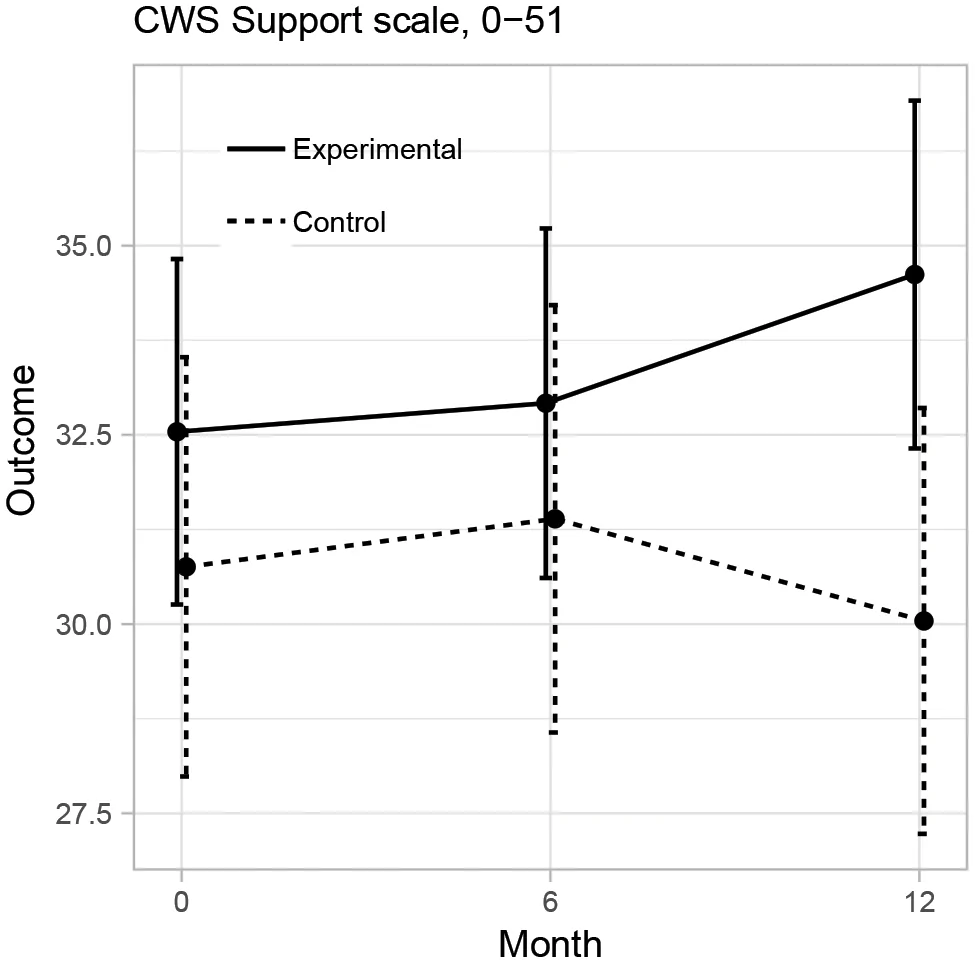
Estimated primary outcome values and 95% confidence intervals from the linear mixed effects model.

**Figure 3** shows the estimated mean, 95% CI and changes of the sum score CWS-B. The variation at center level for this outcome was 1.3% (SD = 1.2%, 1.4%) (intraclass correlation).

In all the sub-scales of CWS-B, there was a tendency towards greater improvement in the intervention compared to the control arm, especially in satisfaction with information and advice, and support from staff at 12 months (d = 0.23, p = 0.12 and d = 0.18, p = 0.17, respectively).

**Figure 4:**
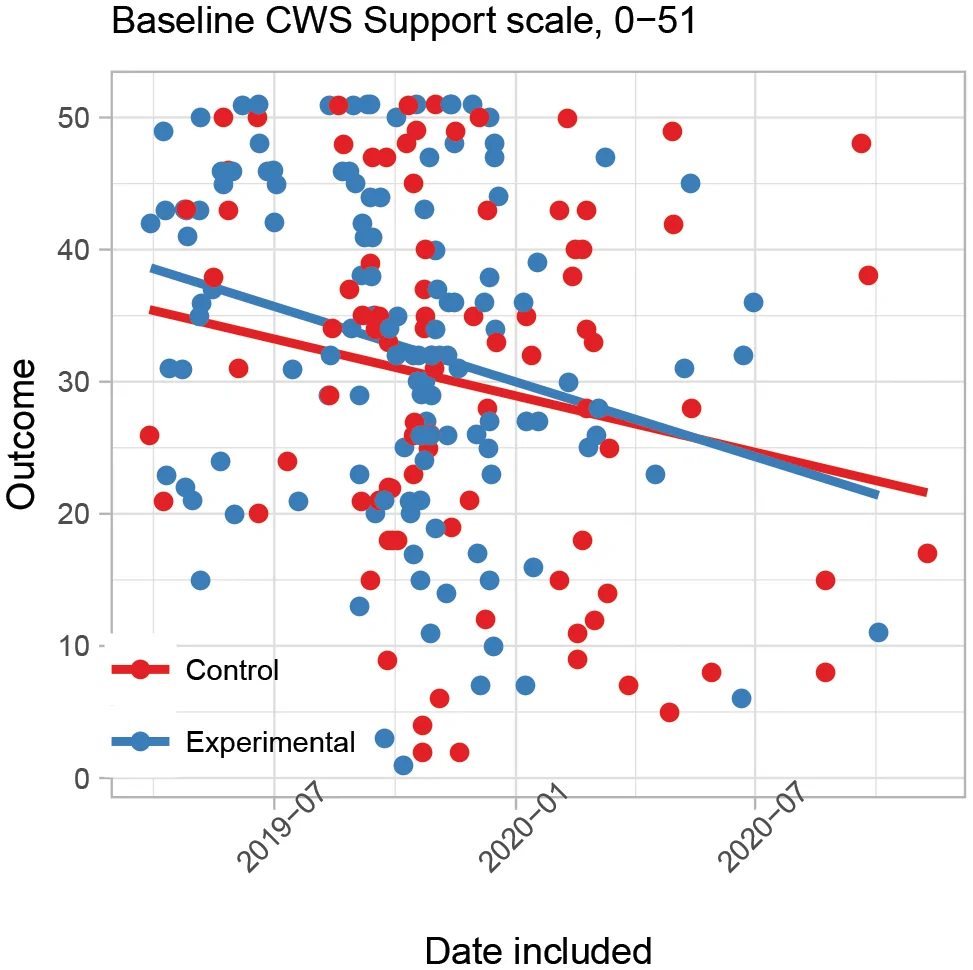
Spread in relatives' satisfaction measured by the CWS sum score in both groups at baseline. Straight lines represent linear regression lines of baseline value by date.

There were large variations in relatives’ responses in both arms at baseline (**Figure 4**). Regression coefficients from the estimated linear mixed effects models are presented in **Supplementary File 3**.

#### 3.3.2 Secondary outcomes: experience of caregiving, expressed emotions and quality of life

Relatives in the intervention arm experienced less patient dependency (ECI) at 12 months (d = -0.23, p = 0.03, **Table 3**). As for CWS-B, there were also large variations in secondary outcomes. For most secondary outcomes, average changes where in the same direction in both arms but non-significantly larger in the intervention arm. Descriptive statistics for all outcomes at baseline, 6 and 12 months are presented in **Supplementary File 4**.

In the sub-group analyzes where we selected only relatives who had received FPE the 12 months after inclusion from the intervention arm and compared with relatives in the control arm who had not received FPE the last 12 months before inclusion, the regression coefficients were in the same direction and showed the same positive trends as the main analyses with larger effect-sizes. Relatives who had received FPE in the intervention group experienced increased information and advice, involvement and support from staff, and a reduced level of patient dependency, compared to the control group at 12 month follow-up (CWS-B and ECI).

## 4 Discussion

### 4.1 Main findings

The relatives in the intervention arm received an increased amount of FI (both FPE and BFIS), including involvement in the patients’ crisis plan, and experienced decreased patient dependency compared to the control arm. There was also a tendency towards increased satisfaction with health service support. Altogether, the IFIP intervention, despite being substantially affected by the pandemic, influenced the clinicians’ family involvement practices sufficiently to have a beneficial effect on relatives’ outcomes and received FI.

The proportion of patient/relative pairs receiving FPE increased considerably in the intervention compared to the control arm, consistent with the findings in our fidelity outcomes study (40). Our findings demonstrate that systematically offering FPE in clinical practice increases the proportion of patients and relatives receiving family involvement in line with recommended treatment for psychotic disorders. This proportion is comparable to the OPUS-study and the TIPS Project of FPE to patients with first-episode psychosis and their relatives (59, 60).

A significantly higher proportion of the relatives in the intervention compared to the control arm had at least one CMHC consultation during the follow-up (80% vs 62%). Most likely, the increase in CMHC consultations in the intervention arm is largely linked to providing BFIS, based on the changes between the arms from baseline to 6 months, which corresponds to the period when the clinical interventions were well underway. This proportion is similar to the study from the OnTrackNY Early Psychosis Services: patients were consistently encouraged to involve their relatives in the treatment, and 73% to 84% of relatives at each measurement point had at least one family consultation (29). The increased proportion of relatives involved in crisis planning as part of the FI, may have contributed to promote the patients’ mental health and reduce the relatives’ stress related to relapse (61).

The COVID-19 pandemic significantly disrupted the delivery of health services. In the intervention units, BFIS and FPE was put on hold, reduced, modified (e.g. digital meetings rather than in-person), terminated earlier than planned, or not initiated at all. Due to the pandemic it was difficult to ensure that the clinical interventions were adherent to the original design in content and quantity, and subsequently to improve FI as intended. The low number of new FPE groups reported after the pandemic outbreak, was also documented in the fidelity study (40). The pandemic led to a sharp decline in CMHC consultations at 12 months in both arms, but more so in the control units. The IFIP intervention focused on better routines, more family-friendly culture, and increased competence and understanding of the significance of family involvement among clinicians (62). At the time of the pandemic outbreak, FI was a more integrated part of the intervention units’ clinical practice and organization, which made them better equipped than the control units to maintain contact with the relatives.

Relatives in the intervention group tended to be more satisfied with support from the health services, mostly in terms of information and advice, and with support from staff. The positive tendencies of support are consistent with implementation studies of FPE in regular clinical practice (43, 63) and with qualitative data published from our study (64). In the latter study clinicians in the intervention arm described that through FPE alliance sessions, they could offer adequate information, guidance and support, helping the relatives to cope with the situation and to support the patient. The clinicians also experienced that family involvement contributed to shared understanding, acknowledgment and mutual dialogue between relatives, patients and clinicians (64). The significance of FI lies in the potential to alleviate the relatives’ sense of isolation in shouldering responsibility for the patient’s situation. Additionally, these interventions play a crucial role in ensuring that the relatives’ efforts are acknowledged by staff and address any negative experiences they may have had with mental health services, as described in previous studies (35, 36, 65).

The level of relatives’ experienced patient dependency was significantly lower in the intervention arm. Dependency is understood as the state in which a person requires the help of others in order to perform daily activities (66). The level of dependency of the care recipient has been shown to be associated with the severity of the illness, and for being the most important predictor of relatives’ burden (29). In FI, the patient’s personal goals for recovery serve as guidance for support and problem solving, through an equal partnership between the patient, relatives and clinician. A strengthened collaboration may have relieved the relatives’ experience of being alone with the responsibility and burden of the patient’s decreased functioning and thereby resulted in a reduced perception of patient dependency. This adds to earlier studies showing strong correlations between family burden and professional support (67, 68).

### 4.2 Limitations

Both contextual factors and factors related to the complex (and pioneering) study design, may have weakened the difference in exposure to FI before and after baseline in the intervention arm, as well as the difference in exposure between the intervention and control arm during the intervention period. In sum, it is likely that this sub-study underreports the effectiveness of the IFIP intervention on relatives’ outcomes such as the degree of satisfaction with health service support.

#### 4.2.1 Inclusion criteria

The inclusion criteria were among the broadest reported in the literature. All available patients in the units were invited to participate in the study, based on ethical and methodological considerations, including to ensure identical eligibility criteria in the two arms. If we had linked the recruitment to receiving or not receiving FI, clinicians in the intervention arm could have prioritized FI to participating patients and relatives, and opposite, clinicians in the control arm could have minimized the FI provided to study participants. This would have affected our principle that neither participation nor non-participation in the research should have any consequences for patient treatment, including to be invited to FI. The inclusion criteria resulted in some patient/relative pairs in the control arm receiving FPE, as well as not all respondents in the intervention arm receiving BFIS or FPE. Thus, the inclusion criteria led to far less differences in exposure to the evidence-based clinical interventions than in standard RCTs or in hybrid studies mainly focusing on clinical effectiveness with less rigorous evaluation of implementation outcomes (69). Finally, the included relatives are next of kin to patients with first-episode psychosis and long-term psychosis. This constitutes a highly diverse group with varied experiences and needs from the healthcare system, thereby resulting in different potentials to improve the relatives’ outcomes through the follow-up period.

#### 4.2.2 Existing family involvement

The IFIP trial focused on increasing the quantity, quality and structure of family involvement in the CMHC units in the intervention arm. There was a lot of contact with the patients’ relatives in both arms at baseline, and in the control group more than half of the relatives had participated in a CMHC consultation during the one-year follow up. To contact relatives for collateral is routine, contact with relatives by phone or consultations is common, and some of the units provided FPE to a limited number of patients before the IFIP trial (27). At all time points, about one in four relatives from both arms reported having at least one consultation with the CMHC. This suggests that some relatives have close contact with the CMHC units, regardless of implementation interventions. With BFIS and FPE as add-on interventions to existing treatment and support, it proved difficult to find significant improvements in relatives’ outcomes.

Independent of the IFIP trial, four teams in the control arm and one team in the intervention arm (27) had recently started Flexible Assertive Community Treatment (FACT) teams. Evidence from Norway indicates that relatives in contact with FACT teams experience greater safety, less stress and more trust compared to other services (70). The establishing of FACT teams may have contributed to a greater proportion of clinically stable patients and to relatives perceiving increased satisfaction with the health services, most notably in the control arm. There may also be other unknown treatments that could have affected the internal validity of the study.

#### 4.2.3 Study design and timeline challenges

The IFIP trial provides important insights into possible biases and time frame challenges that may be encountered when attempting to objectively assess implementation outcomes and effectiveness outcomes for patients’ and relatives’ at the same time. For example, recruitment of patients and relatives and training in the clinical interventions started simultaneously, and the recruitment period was long and prolonged (19 months), partly due to the Covid-19 pandemics. Thus, many of the patients and relatives in the intervention arm already had initiated BFIS and FPE when being recruited, probably affecting the baseline data. The CWS Support sum had a higher value in the intervention arm compared to the control arm, which may have resulted in reduced differences in change over time. Limited resources as well as a relatively short project period was an obstacle for the CMCH units when aiming to provide BFIS and FPE to all patients and relatives. Our fidelity results show that the intervention units rapidly completed training, established implementation teams and appointed a family coordinator, while the penetration of FI had a slower and more gradual increase (40). This reflects that time is required for organizational and procedural changes to increase the amount of FI in everyday clinical practice, which subsequently can improve the relatives’ outcomes (71, 72). The responsive design of the study, with communication and possible engagement for FI improvements before recruitment of the units, and the baseline fidelity measurement before randomization of the included units, may also have influenced the units’ use of family involvement in both arms before inclusion of patients and relatives.

Further discussions and research is needed to optimize research designs and explore issues related to timeframes and time points in longitudinal implementation studies measuring both implementation outcomes and clinical effectiveness (41). Possible mitigating factors for future research may be more strict inclusion and exclusion criteria for both the health service units and the patients/relatives, and to make baseline measurements in comparable groups of patients and relatives before randomization of the units.

### 4.3 Strengths

The response rates at the follow-ups were notably high (**Figure 1**), especially considering the life situations of the included relatives. Another strength lies in the results from the subgroup analyses, consistently reinforcing the main findings concerning relatives’ positive outcomes. Moreover, the naturalistic design contributed to knowledge that is relevant to ordinary clinical practice, which is highly needed in mental health services (73). An important implementation strategy was the “whole ward approach”, to increase the probability that all patients with psychotic disorders and their relatives were offered some kind of systematic FI. Both BFIS and FPE were provided by ordinary clinical staff, as one of several strategies to enhance sustainment of high-quality FI after the project period. Finally, the IFIP trial is a unique large-scale project to implement basic family involvement and FPE, with fidelity measurements (27, 40), qualitative data (62, 64, 74, 75), and health register data which complements the quantitative data from relatives presented in this paper and strengthens our understanding of FI.

### 4.4 Implications for mental health services

Family involvement is consistently recommended in treatment guidelines worldwide for individuals with a psychotic disorder, which should entail that relatives receive continuous information, guidance, and support from mental health services (30). Therefore, it is crucial that all CMHC units ensure routines, knowledge, and competence among clinicians to systematically offer FI tailored to the needs of patients and their families. The IFIP-trial, with this sub-study on relatives’ outcomes and the fidelity outcome study on the cluster-level, has demonstrated the possibility of enhancing FI on a large scale with positive outcomes for relatives. The forthcoming patient outcome study (in process) aligns with the results in the current study. Thus, it is possible and feasible for CMHCs to implement and integrate FI for patients with psychotic disorders and their families in an evidence-based, safe, and effective manner. Our approach to implement FI seems to reduce relatives’ needs for other support and more *ad hoc* services. A systematic implementation of FI may overall be the most sustainable and cost-efficient approach. Future research and publications will examine the costs and sustainability associated with implementing the IFIP intervention in the mental health services.

## 5 Conclusions

This sub-study, despite being affected by the pandemic, makes a substantial contribution to understand the effect on relatives outcomes when implementing family involvement in CMHC units for patients with psychotic disorders. The relatives received an increased amount of FI, including involvement in crisis planning, and experienced a decreased level of patient dependency. The improved support from the CMHC units may have relieved the relatives’ experience of responsibility and caregiver burden. Our data confirm the potential the mental health services have through systematically offering family involvement in regular clinical practice.

## Data Availability

The original contributions presented in the study are included in the article/**Supplementary Material**. Further inquiries can be directed to the corresponding author.

## Supplementary material

Supplementary File 1 Report on Consolidated Standards of Reporting Trials (CONSORT) statement 2010: extension to cluster randomized trials.

Supplementary File 2 Timeline.

Supplementary File 3 Regression coefficients for primary and secondary outcomes.

Supplementary File 4 Descriptive statistics for primary and secondary outcomes.
