# Correction: Implementation of guidelines on Family involvement for persons with Psychotic disorders: a pragmatic cluster randomized trial. Effect on relatives’ outcomes and family interventions received

**DOI:** 10.3389/fpsyt.2026.1983658

**Published:** 2026-09-14

**Authors:** Irene Norheim, Reidar Pedersen, Maria Lie Selle, Jan Ivar Røssberg, Lars Hestmark, Kristin Sverdvik Heiervang, Torleif Ruud, Vilde Maria Åsholt, Kristiane Myckland Hansson, Paul Møller, Roar Fosse, Maria Romøren

**Affiliations:** 1 Department of Mental Health Research and Development, Division of Mental Health and Addiction, Vestre Viken Hospital Trust, Drammen, Norway; 2 Centre for Medical Ethics, Institute of Health and Society, University of Oslo, Oslo, Norway; 3 Health Services Research Unit, Akershus University Hospital, Lørenskog, Norway; 4 Section for Treatment Research, Division of Mental Health and Addiction, Oslo University Hospital, Oslo, Norway; 5 Institute of Clinical Medicine, Faculty of Medicine, University of Oslo, Oslo, Norway; 6 Division of Mental Health Services, Akershus University Hospital, Lørenskog, Norway

**Keywords:** schizophrenia, severe mental illness, caregivers, family intervention, family psychoeducation, evidence based practices, mental health services

Affiliation 2 “Centre for Medical Ethics, Institute of Health and Society, University of Oslo, Oslo, Norway” was omitted for author “Irene Norheim”. This affiliation has now been correctly added by her name.

The original version of this article has been updated.

