# Supplementary material for: Implementation of guidelines on Family Involvement for persons with Psychotic disorders: a pragmatic cluster randomized trial. Effect on relatives’ outcomes and family interventions received
Source: Front Psychiatry. 2024 May 24;15:1381007. doi: 10.3389/fpsyt.2024.1381007 (PMC11157113; doi:10.3389/fpsyt.2024.1381007)
Supplement: Supplementary File 1 — Report on Consolidated Standards of Reporting Trials (CONSORT) statement 2010: extension to cluster randomized trials. [file DataSheet_1.zip › Supplementary file 2. Timeline.pdf]

Supplementary file 2: Timeline for the IFIP-trial project activities.

**Activity Month**	0[1]	1	2	3	4	5	6	7	8	9	10	11	12	13	14	15	16	17	18	19	20	21	22	23	24	25	26	27	28	29	30	31
Fidelity measure-ments interv. arm[2]	x	x											x	x					x	x					x	x						
Fidelity measure-ments control arm	x	x																														
Randomisation			x																													
Inclusion of patients and relatives					x[3]	x	x	x	x	x	x	x	x	x	x	x	x	x*	x*	x*	x*	x*	x*									
Follow-up at 6 and 12 month											x	x	x	x	x	x	x	x	x	x	x	x	x	x*	x*	x*	x*	x*	x*			
IFIP intervention in intervention arm				x	x	x	x	x	x	x	x	x	x	x	x	x	x	x	x	x	x	x	x	x	x							
IFIP intervention in control arm																												x	x	x	x	x
**External factors**																																
Sars-COV-19 pandemic																	x	x	x	x	x	x	x	x	x	x	x	x	x	x	x	x

* Prolonged due to the Sars-COV-19 pandemic.

[1] November 2018

[2] Measure of the level of implementation.

[3] At baseline, the relatives reported family involvement and support services participation “the last 12 months” and also participation before that, called “previous participation”.
