# Supplementary material for: Implementation of guidelines on Family Involvement for persons with Psychotic disorders: a pragmatic cluster randomized trial. Effect on relatives’ outcomes and family interventions received
Source: Front Psychiatry. 2024 May 24;15:1381007. doi: 10.3389/fpsyt.2024.1381007 (PMC11157113; doi:10.3389/fpsyt.2024.1381007)
Supplement: Supplementary File 1 — Report on Consolidated Standards of Reporting Trials (CONSORT) statement 2010: extension to cluster randomized trials. [file DataSheet_1.zip › Supplementary file 3. Regression coefficient.pdf]

Supplementary file 3. Regression coefficients for primary and secondary outcomes.

Outcome	Parameter	Estimate	Standard error	95% CI lower	95% CI upper	p value
**Primary outcome**						
CWS_support_sum	Intercept	30,76	1,41	27,99	33,53	<0,01
N = 204	Arm at baseline	1,79	1,83	-1,80	5,37	0,33
N intervention = 122	Time 6mo	0,64	1,25	-1,82	3,09	0,61
N control = 82	Time 12mo	-0,72	1,25	-3,16	1,73	0,57
	Time 6mo x Arm	-0,26	1,61	-3,42	2,91	0,87
	Time 12mo x Arm	2,79	1,62	-0,38	5,96	0,08
CWS_information	Intercept	14,13	0,69	12,78	15,47	<0,01
N = 204	Arm at baseline	0,81	0,88	-0,92	2,54	0,36
N intervention = 122	Time 6mo	0,40	0,63	-0,84	1,64	0,53
N control = 82	Time 12mo	0,07	0,62	-1,14	1,27	0,91
	Time 6mo x Arm	0,05	0,81	-1,53	1,63	0,96
	Time 12mo x Arm	1,09	0,79	-0,46	2,64	0,17
CWS_involvement	Intercept	3,14	0,22	2,71	3,56	<0,01
N = 203	Arm at baseline	0,44	0,28	-0,11	0,99	0,12
N intervention = 121	Time 6mo	0,22	0,22	-0,20	0,65	0,30
N control = 82	Time 12mo	0,26	0,21	-0,14	0,66	0,21
	Time 6mo x Arm	-0,20	0,28	-0,75	0,34	0,46
	Time 12mo x Arm	0,03	0,26	-0,49	0,55	0,91
CWS_support_from_staff	Intercept	13,30	0,63	12,06	14,53	<0,01
N = 200	Arm at baseline	0,83	0,82	-0,78	2,44	0,31
N intervention = 119	Time 6mo	0,32	0,63	-0,91	1,54	0,61
N control = 81	Time 12mo	-0,92	0,63	-2,15	0,32	0,15
	Time 6mo x Arm	-0,53	0,80	-2,11	1,05	0,51
	Time 12mo x Arm	1,29	0,82	-0,32	2,91	0,12
**Secondary outcomes**						
CWS_Additional question_Satisfaction	Intercept	1,70	0,11	1,49	1,92	<0,01
N = 201	Arm at baseline	0,15	0,15	-0,14	0,43	0,32
N intervention = 120	Time 6mo	0,07	0,12	-0,17	0,30	0,59
N control = 81	Time 12mo	-0,04	0,12	-0,27	0,19	0,72
	Time 6mo x Arm	-0,05	0,16	-0,36	0,25	0,74
	Time 12mo x Arm	0,20	0,15	-0,11	0,50	0,21
ECI_Negative_scale	Intercept	70,54	3,73	63,22	77,85	<0,01
N = 210	Arm at baseline	0,39	4,86	-9,12	9,91	0,94
N intervention = 124	Time 6mo	-0,32	2,40	-5,02	4,38	0,89
N control = 86	Time 12mo	-2,57	2,32	-7,11	1,98	0,27
	Time 6mo x Arm	-2,96	3,10	-9,04	3,11	0,34
	Time 12mo x Arm	-3,26	3,02	-9,18	2,66	0,28
ECI_Positive_scale	Intercept	27,55	0,90	25,78	29,31	<0,01
N = 210	Arm at baseline	-1,50	1,17	-3,80	0,80	0,20
N intervention = 124	Time 6mo	-1,07	0,69	-2,43	0,29	0,12
N control = 86	Time 12mo	-1,91	0,67	-3,22	-0,60	0,00
	Time 6mo x Arm	-1,00	0,89	-2,74	0,75	0,26
	Time 12mo x Arm	-0,65	0,86	-2,34	1,05	0,45
ECI_Difficult_behaviours	Intercept	9,04	0,74	7,60	10,47	<0,01
N = 210	Arm at baseline	0,14	0,96	-1,73	2,02	0,88
N intervention = 124	Time 6mo	0,40	0,54	-0,66	1,46	0,46
N control = 86	Time 12mo	0,42	0,54	-0,63	1,48	0,43
	Time 6mo x Arm	-0,33	0,70	-1,71	1,04	0,64
	Time 12mo x Arm	-0,31	0,70	-1,68	1,06	0,65
ECI_Negative_symptoms	Intercept	9,19	0,64	7,94	10,44	<0,01
N = 210	Arm at baseline	1,34	0,83	-0,29	2,96	0,11
N intervention = 124	Time 6mo	0,37	0,49	-0,59	1,32	0,45
N control = 86	Time 12mo	0,20	0,46	-0,71	1,11	0,67
	Time 6mo x Arm	-0,74	0,63	-1,98	0,50	0,24
	Time 12mo x Arm	-0,86	0,61	-2,05	0,34	0,16
ECI_Stigma	Intercept	5,08	0,40	4,31	5,86	<0,01
N = 210	Arm at baseline	0,12	0,52	-0,89	1,13	0,82
N intervention = 124	Time 6mo	0,24	0,33	-0,40	0,88	0,46

N control = 86	Time 12mo	0,21	0,32	-0,41	0,84	0,50
	Time 6mo x Arm	0,03	0,42	-0,80	0,86	0,95
	Time 12mo x Arm	0,05	0,41	-0,76	0,86	0,90
ECI_Problems_with_services	Intercept	11,63	0,72	10,22	13,03	<0,01
N = 210	Arm at baseline	-1,19	0,93	-3,02	0,64	0,20
N intervention = 124	Time 6mo	-1,18	0,58	-2,33	-0,04	0,04
N control = 86	Time 12mo	-0,98	0,55	-2,06	0,11	0,08
	Time 6mo x Arm	0,05	0,75	-1,41	1,51	0,95
	Time 12mo x Arm	-0,58	0,72	-1,99	0,84	0,42
ECI_Effects_on_family	Intercept	8,57	0,62	7,35	9,79	<0,01
N = 210	Arm at baseline	-0,38	0,81	-1,97	1,20	0,63
N intervention = 124	Time 6mo	-0,47	0,42	-1,30	0,36	0,27
N control = 86	Time 12mo	-0,49	0,41	-1,29	0,32	0,24
	Time 6mo x Arm	0,35	0,55	-0,73	1,43	0,53
	Time 12mo x Arm	0,26	0,54	-0,81	1,31	0,64
ECI_Need_to_backup	Intercept	6,70	0,56	5,61	7,79	<0,01
N = 210	Arm at baseline	0,32	0,72	-1,10	1,73	0,66
N intervention = 124	Time 6mo	0,10	0,39	-0,67	0,87	0,80
N control = 86	Time 12mo	-0,61	0,38	-1,36	0,14	0,11
	Time 6mo x Arm	-1,00	0,51	-2,00	0,01	0,05
	Time 12mo x Arm	-0,44	0,50	-1,41	0,53	0,37
ECI_Dependency	Intercept	9,19	0,46	8,29	10,08	<0,01
N = 210	Arm at baseline	0,36	0,60	-0,81	1,53	0,54
N intervention = 124	Time 6mo	0,28	0,34	-0,38	0,93	0,41
N control = 86	Time 12mo	-0,37	0,33	-1,02	0,28	0,27
	Time 6mo x Arm	-0,81	0,44	-1,67	0,05	0,06
	Time 12mo x Arm	-0,96	0,43	-1,81	-0,11	0,03
ECI_Loss	Intercept	11,15	0,57	10,04	12,27	<0,01
N = 210	Arm at baseline	-0,31	0,74	-1,76	1,14	0,67
N intervention = 124	Time 6mo	-0,08	0,39	-0,84	0,69	0,85
N control = 86	Time 12mo	-0,93	0,38	-1,66	-0,19	0,01
	Time 6mo x Arm	-0,38	0,51	-1,37	0,61	0,45
	Time 12mo x Arm	-0,48	0,49	-1,44	0,48	0,33
ECI_Positive_personal_experiences	Intercept	14,24	0,59	13,09	15,40	<0,01
N = 210	Arm at baseline	-0,95	0,77	-2,45	0,56	0,22
N intervention = 124	Time 6mo	-0,29	0,46	-1,19	0,62	0,53
N control = 86	Time 12mo	-1,03	0,45	-1,92	-0,14	0,02
	Time 6mo x Arm	-0,98	0,60	-2,15	0,19	0,10
	Time 12mo x Arm	-0,52	0,59	-1,67	0,63	0,37
ECI_Good_aspects_of_relationship	Intercept	13,30	0,44	12,45	14,16	<0,01
N = 210	Arm at baseline	-0,55	0,57	-1,66	0,56	0,33
N intervention = 124	Time 6mo	-0,72	0,36	-1,42	-0,01	0,05
N control = 86	Time 12mo	-0,90	0,35	-1,58	-0,22	0,01
	Time 6mo x Arm	-0,11	0,46	-1,02	0,80	0,81
	Time 12mo x Arm	-0,11	0,45	-0,99	0,78	0,81
FQ_Emotional overinvolvement	Intercept	21,64	0,59	20,48	22,80	<0,01
N = 208	Arm at baseline	-0,07	0,77	-1,58	1,44	0,93
N intervention = 122	Time 6mo	0,63	0,42	-0,19	1,45	0,13
N control = 86	Time 12mo	0,47	0,40	-0,32	1,26	0,24
	Time 6mo x Arm	0,81	0,55	-0,26	1,89	0,14
	Time 12mo x Arm	0,32	0,53	-0,71	1,35	0,55
FQ_Criticism	Intercept	16,65	0,62	15,44	17,87	<0,01
N = 208	Arm at baseline	0,72	0,80	-0,86	2,29	0,37
N intervention = 124	Time 6mo	0,32	0,47	-0,61	1,25	0,50
N control = 84	Time 12mo	-0,13	0,45	-1,01	0,76	0,78
	Time 6mo x Arm	0,19	0,60	-1,00	1,37	0,75
	Time 12mo x Arm	0,27	0,59	-0,88	1,43	0,64
CarerQoL_VAS	Intercept	6,48	0,20	6,10	6,86	<0,01
N = 210	Arm at baseline	0,00	0,25	-0,50	0,50	1,00
N intervention = 124	Time 6mo	-0,21	0,16	-0,53	0,11	0,20
N control = 86	Time 12mo	-0,11	0,16	-0,42	0,20	0,48
	Time 6mo x Arm	0,19	0,21	-0,22	0,60	0,36
	Time 12mo x Arm	0,20	0,21	-0,20	0,61	0,33
