# Supplementary material for: Implementation of guidelines on Family Involvement for persons with Psychotic disorders: a pragmatic cluster randomized trial. Effect on relatives’ outcomes and family interventions received
Source: Front Psychiatry. 2024 May 24;15:1381007. doi: 10.3389/fpsyt.2024.1381007 (PMC11157113; doi:10.3389/fpsyt.2024.1381007)
Supplement: Supplementary File 1 — Report on Consolidated Standards of Reporting Trials (CONSORT) statement 2010: extension to cluster randomized trials. [file DataSheet_1.zip › Supplementary file 4. Descriptive statistics all outcomes.pdf]

Supplementary file 4. Descriptive statistics for all outcomes at baseline, 6 and 12 months follow-up.

		BASELINE						6 MONTHS FOLLOW-UP				12 MONTHS FOLLOW-UP			
		Combined		Control		Intervention		Control		Intervention		Control		Intervention	
Primary outcome	Range	N	Mean (SD)	N	Mean (SD)	N	Mean (SD)	N	Mean (SD)	N	Mean (SD)	N	Mean (SD)	N	Mean (SD)
**CWS Support sum[a]**	**0-51↑**	**222**	**31.56 (13.17)**	**92**	**29.89 (13.83)**	**130**	**32.74 (12.6)**	**73**	**31.22 (13.61)**	**115**	**33.04 (12.44)**	**80**	**30.31 (13.55)**	**120**	**34.73 (11.98)**
CWS Information[a]	0-24↑	221	14.51 (6.43)	91	13.84 (6.62)	130	14.98 (6.27)	73	14.44 (6.58)	114	15.44 (6.08)	79	14.46 (6.35)	120	16.15 (5.55)
CWS Involvement[a]	0-6↑	220	3.39 (1.98)	91	3.1 (2.13)	129	3.6 (1.85)	72	3.42 (2.07)	114	3.64 (1.94)	81	3.43 (1.97)	120	3.92 (1.77)
CWS Support from staff[a]	0-21↑	215	13.67 (5.76)	90	12.91 (6.4)	125	14.21 (5.22)	70	13.64 (5.77)	113	13.96 (5.56)	77	12.7 (6.09)	116	14.59 (5.42)
Secondary outcomes	Range	N	Mean (SD)	N	Mean (SD)	N	Mean (SD)	N	Mean (SD)	N	Mean (SD)	N	Mean (SD)	N	Mean (SD)
CWS Additional question[a]	0-3↑	218	1.8 (1)	92	1.68 (1.03)	126	1.89 (0.98)	70	1.8 (1.06)	113	1.88 (1.04)	78	1.68 (1.05)	117	2.02 (0.88)
ECI Negative scale[b]	0-208↓	230	70.67 (31.97)	96	70.74 (32.14)	134	70.63 (31.98)	76	69.97 (35.61)	116	67.78 (33.76)	83	67.75 (35.16)	120	64.14 (36.48)
ECI Positive scale[b]	0-56↑	230	26.52 (7.9)	96	27.4 (8.08)	134	25.9 (7.73)	76	26.53 (8.22)	116	23.93 (8.34)	83	26.01 (8.39)	120	23.33 (9.07)
ECI Difficult behaviours[b]	0-32↓	230	9.12 (6.4)	96	9 (6.76)	134	9.2 (6.15)	76	9.63 (7.4)	116	9.33 (6.59)	83	9.31 (7.01)	120	9.07 (6.91)
ECI Negative symptoms[b]	0-24↓	230	10.13 (5.51)	96	9.45 (5.27)	134	10.61 (5.64)	76	9.66 (6.11)	116	10.21 (6)	83	9.39 (5.69)	120	9.68 (6.32)
ECI Stigma[b]	0-20↓	230	5.06 (3.32)	96	4.94 (3.54)	134	5.15 (3.17)	76	5.38 (3.59)	116	5.54 (3.95)	83	5.23 (3.91)	120	5.38 (3.86)
ECI Problems with services[b]	0-32↓	230	10.84 (6.35)	96	11.67 (6.38)	134	10.25 (6.28)	76	10.32 (6.6)	116	9.22 (6.44)	83	10.59 (6.96)	120	8.77 (6.91)
ECI Effects on family[b]	0-28↓	230	8.35 (5.69)	96	8.45 (5.88)	134	8.28 (5.57)	76	7.93 (5.79)	116	8.05(5.48)	83	8.07 (6.1)	120	7.84(5.65)
ECI Need to backup[b]	0-24↓	230	6.93 (5.09)	96	6.85 (5.29)	134	6.99 (4.96)	76	6.76 (5.62)	116	6.06 (4.87)	83	6.07 (5.13)	120	5.91 (5.08)
ECI Dependency[b]	0-20↓	230	9.4 (4.09)	96	9.27 (4.17)	134	9.49 (4.05)	76	9.39 (4.29)	116	9 (4.12)	83	8.87 (4.29)	120	8.13 (4.32)
ECI Loss[b]	0-28↓	230	10.85 (5.06)	96	11.11 (5.01)	134	10.66 (5.11)	76	10.89 (5.2)	116	10.37 (5.33)	83	10.22 (5.02)	120	9.36 (5.46)
ECI Positive personal exp.[b]	0-32↑	230	13.51 (5.35)	96	14.06 (5.44)	134	13.12 (5.27)	76	14.03 (5.6)	116	12.05 (5.38)	83	13.34 (5.73)	120	11.62 (5.48)
ECI Good aspects relations[b]	0-24↑	230	13.01 (3.85)	96	13.33 (3.96)	134	12.78 (3.77)	76	12.5 (4.1)	116	11.88 (3.97)	83	12.67 (3.86)	120	11.72 (4.39)
FQ EOI[c]	10-40↓	228	21.47 (4.69)	96	21.64 (4.52)	132	21.36 (4.83)	77	22.08 (5.38)	113	23 (5.76)	83	21.99 (5.34)	116	22.03 (6.22)
FQ Criticism[c]	10-40↓	226	17.08 (5.28)	93	16.66 (5.02)	133	17.38 (5.44)	74	16.93 (5.81)	114	17.89 (5.64)	83	16.53 (5.5)	119	17.29 (6.1)
CarerQoL-VAS[d]	0-10↑	230	6.57 (1.83)	96	6.55 (1.74)	134	6.57 (1.91)	73	6.23 (1.78)	116	6.4 (1.76)	84	6.38 (1.83)	121	6.6 (1.78)
a) CarerQoL [d]	0-2↑	226	1.44 (0.59)	95	1.52 (0.62)	131	1.39 (0.56)	74	1.35 (0.58)	115	1.34 (0.61)	83	1.37 (0.56)	119	1.34 (0.59)
b) CarerQoL [d]	0-2↑	229	1.52 (0.54)	96	1.58 (0.54)	133	1.48 (0.54)	76	1.49 (0.6)	117	1.43 (0.58)	84	1.48 (0.61)	121	1.47 (0.61)
c) CarerQoL [d]	0-2↑	230	1.48 (0.57)	96	1.49 (0.6)	134	1.47 (0.56)	76	1.39 (0.57)	117	1.41 (0.57)	84	1.38 (0.6)	121	1.45 (0.61)
d) CarerQoL [d]	0-2↑	229	1.59 (0.58)	96	1.57 (0.59)	133	1.6 (0.58)	74	1.57 (0.58)	116	1.53 (0.57)	84	1.56 (0.52)	121	1.55 (0.59)
e) CarerQoL [d]	0-2↑	230	1.76 (0.48)	96	1.75 (0.5)	134	1.77 (0.46)	74	1.73 (0.48)	116	1.78 (0.49)	84	1.77 (0.45)	121	1.79 (0.46)
f) CarerQoL [d]	0-2↑	228	0.91 (0.71)	94	0.91 (0.73)	134	0.9 (0.69)	73	0.84 (0.69)	116	0.87 (0.7)	84	0.79 (0.71)	121	0.83 (0.72)
g) CarerQoL[d]	0-2↑	230	1.4 (0.64)	96	1.43 (0.63)	134	1.39 (0.65)	74	1.38 (0.63)	116	1.44 (0.65)	84	1.42 (0.59)	121	1.48 (0.66)

Presented descriptive statistics are Mean (SD). Range: ↓= Higher value, negative outcome, ↑= Higher value, positive outcome.

[a]Carer Well-being and Support questionnaire Support scale (CWS-part B). CWS Information = Information and advice for carers. CWS Involvement = Involvement in treatment and care planning CWS Support from staff = CWS Support from medical and/or care staff. CWS Additional question = A single question about satisfaction with health services from an earlier version of CWS-B: *Overall. how satisfied are you with the support you receive to help you in your role as a relative?* [b]The Experience of Care-giving inventory questionnaire (ECI). Subscales. [c]The Family questionnaire (FQ). EOI = Emotional overinvolvement. [d]The Care Related Quality of Life questionnaire (Carer QoL). Carer QoL-VAS (Visual Analogue Scale), and each question: a) Fulfilment with carrying out my care tasks, b) Relational problems with the care receiver, c) Problems with my own mental health, d) Problems combining my care tasks with my daily activities, e) Financial problems because of my care tasks, f) Support with carrying out my care tasks, when I need it, g) Problems with my own physical health.
